# Health Status, Physical Disability, and Obesity Among Adult Mississippians With Chronic Joint Symptoms or Doctor-Diagnosed Arthritis: Findings From the Behavioral Risk Factor Surveillance System, 2003

**Published:** 2008-06-15

**Authors:** Nadine T James, Carl W Miller, Peter J Fos, Lei Zhang, Peggy Wall, Cindy Welch

**Affiliations:** School of Nursing, University of Southern Mississippi; College of Health, The University of Southern Mississippi, Hattiesburg, Mississippi; Health Services Data Unit, Mississippi Department of Health, Jackson, Mississippi; Health Services Data Unit, Mississippi Department of Health, Jackson, Mississippi; Arthritis Foundation, Mississippi Chapter, Jackson, Mississippi; School of Nursing, The University of Southern Mississippi, Hattiesburg, Mississippi

## Abstract

**Introduction:**

The purpose of this study was to analyze 2003 Mississippi Behavioral Risk Factor Surveillance System (BRFSS) data to describe the health of Mississippians with arthritis or chronic joint pain. For this study, we made statistical estimates of the extent of arthritis burden among the respondents and delineated measurable differences in sociodemographic factors, health status, and the prevalence of associated risk factors. Our findings compare health-related quality of life, physical activity, and key demographic characteristics and obesity rates, controlling for differences among the subgroups by age, sex, educational attainment, income, and race/ethnicity.

**Methods:**

Respondents to Mississippi’s 2003 BRFSS were assigned to 1 of 5 distinct and mutually exclusive subgroups: 1) those with intermittent joint symptoms (IJS), 2) those with chronic joint symptoms (CJS), 3) those with doctor-diagnosed arthritis without CJS (DDA − CJS), 4) those with doctor-diagnosed arthritis with chronic joint symptoms (DDA + CJS), and 5) those with no joint symptoms (NJS). To determine the prevalence of arthritis and the continuum of disease progression, we compared the health-related quality of life, physical activity, and obesity of the respondents.

**Results:**

Respondents with DDA + CJS were older than those with NJS (mean age, 57.1 years vs 38.7 years); they were more likely to be female (60.5% vs 51.7%), to have a high school diploma or less education (59.3% vs 45.4%), to be in fair to poor health (odds ratio [OR], 10.0), to be physically inactive (OR, 2.7), and to be overweight or obese (OR, 2.5).

**Conclusion:**

Health status, physical disability, and weight control may be substantially improved through heightened levels of physical activity. However, in spite of the potential for marked improvement, adult Mississippians, especially those clients with DDA + CJS, remain reluctant to commit to exercise regimens. Findings from this study suggest a need to encourage Mississippians with DDA + CJS to engage in some regular physical activity, which could reduce the damaging effects of disease and improve their health. Increasing the health care resources earmarked for arthritis self-help and physical activity programs is one potential avenue to address the problem.

## Introduction

Arthritis is the most common disease among America's adult population (1) and among Mississippi's estimated 2.9 million residents (2). Approximately 31.4% of Mississippians have arthritis, which causes extensive physical disability and pain among this undertreated population (3). Unaddressed medical needs are, in part, the consequence of deficiencies in Mississippi's health care continuum. Despite the state's extensive array of health care providers, community health centers, rural health clinics, hospitals, nursing homes, and other health services, Mississippians have been determined by the U.S. Department of Health and Human Services to be medically underserved, especially in care for those with rheumatic disease (4).

Even though arthritis and chronic joint symptoms affect 1 in 3 Mississippians — a ratio that is projected to increase as the state's population ages (5) — efforts to manage these disease symptoms and their progression have had limited success (6). Independent studies have shown that for those with arthritis and chronic joint symptoms, attendant pain can be reduced, function and quality of life can be improved, and disability can be delayed through physical activity and management of weight (7-9). However, no statistically significant progress in reaching related national health objectives for 2010 (10) has been made in the state (6).

Clear deficits in health-related quality of life, illness, and physical activity among respondents with DDA + CJS (11) have been documented through analyses of Behavioral Risk Factor Surveillance System (BRFSS) data.

The purpose of our study was to describe arthritis prevalence among the respondents and to delineate measurable differences in sociodemographic factors, health status, and the prevalence of associated risk factors. To do so, we analyzed 2003 data from the Mississippi BRFSS to compare health status, activity limitation, obesity, and disease management among 5 distinct and mutually exclusive subgroups of respondents to Mississippi's 2003 BRFSS: those with intermittent joint symptoms (IJS), chronic joint symptoms (CJS), doctor-diagnosed arthritis without CJS (DDA − CJS), DDA + CJS, and no joint symptoms (NJS). The subgroup with no joint symptoms was considered the reference group. We compared the subgroups' health-related quality of life, physical activity, key demographic characteristics, and obesity rates, controlling for differences in age, sex, educational attainment, income, and race/ethnicity.

## Methods

The BRFSS is a state-based, random-digit–dialed telephone survey of the U.S. civilian, noninstitutionalized population aged ≥18 years. It is administered annually in all 50 states, the District of Columbia, and 3 territories (Guam, Puerto Rico, and the U.S. Virgin Islands). Because BRFSS respondents are a disparate group with and without current joint symptoms, BRFSS data permit investigators to examine, among other topics, the epidemiology of arthritis and its associated risk factors.

Data from the 2003 BRFSS were collected and analyzed as part of a collaborative effort between the Centers for Disease Control and Prevention (CDC) and the Mississippi State Department of Health. Uniform data on key preventive health practices and risk behaviors of the population aged ≥18 years that live in Mississippi households were collected from 4422 respondents. The median state response rate was 42.7%. Poststratification weights were employed to correct inherent biases in the study design, to adjust for differences in probability of selection and nonresponse, and to derive representative population-based estimates of risk behavior prevalence.

### Chronic joint symptoms and doctor-diagnosed arthritis

The relationship between the duration of joint symptoms and doctor-diagnosed arthritis was assessed by using responses to 4 survey questions: 1) "During the past 30 days, have you had any symptoms of pain, aching, or stiffness in or around a joint?"; 2) "Did your joint symptoms first begin more than 3 months ago?"; 3) "Have you ever seen a doctor or other health professional for these joint symptoms?"; and 4) "Have you been told by a doctor you have some form of arthritis, rheumatoid arthritis, gout, lupus, or fibromyalgia?" On the basis of their responses, respondents were placed into 1 of 5 subgroups corresponding to CDC's case definition for arthritis (Table 1).

### Sociodemographic characteristics, health status, physical activity, and obesity

Age, sex, race/ethnicity, educational attainment, and annual household income (<$35,000 vs ≥$35,000) were evaluated within each subgroup. Health status was assessed by analyzing responses to 3 related questions: 1) "Would you say that in general your health is excellent, very good, good, fair, or poor?"; 2) "Now thinking about your physical health, which includes physical illness and injury, for how many days during the past 30 days was your physical health not good?"; and 3) "Now thinking about your mental health, which includes stress, depression, and problems with emotions, for how many days during the past 30 days was your mental health not good?" Physical activity was evaluated by means of a 3-level variable that categorized respondents by their self-reported physical activity level: 1) they met recommendations for moderate or vigorous physical activity; 2) they did not meet recommendations for levels of physical activity; or 3) they were doing no moderate or vigorous physical activity. Finally, respondents were categorized as body mass index [BMI] <25.0 kg/m^2^, BMI 25.0 kg/m^2^ to 29.9 kg/m^2^, or BMI ≥30.0 kg/m^2^.

### Statistical analyses

SAS version 9.2 (SAS Institute Inc, Cary, North Carolina) and SUDAAN version 9.01 (Research Triangle Institute, Research Triangle Park, North Carolina) were used to conduct descriptive, bivariate, and multinomial regression analyses on the complex, weighted 2003 BRFSS data. Appropriate sampling weights were applied in all point estimations. Additionally, adjusted ORs and 95% confidence intervals (CI) were derived by using SUDAAN modeling techniques that employed the Taylor series method. The attendant multinomial regression models indicated that respective odds ratios controlled for age, sex, race/ethnicity, and educational attainment and predicted likely effect sizes of the independent variables of interest.

## Results

The state's estimated population comprised people reporting CJS (19%), doctor-diagnosed arthritis (27%), and intermittent (but not chronic) joint symptoms (7%). Doctor-diagnosed arthritis was further divided into those with CJS (25%) and those without (2%). Slightly less than half (47%) of all Mississippians reported no arthritis or CJS; however, the number of those with CJS, arthritis, or both is expected to increase dramatically (Figure) as the population ages.

**Figure.**
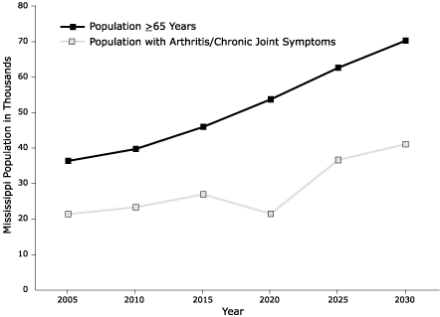
Estimates of Population Aged ≥ 65 Years and Number With Arthritis or Chronic Joint Symptoms, by Year —Behavioral Risk Factor Surveillance System, United States.

**Table:** 

Sociodemographic comparisons among the 5 subgroups (Table 2) reveal that respondents with DDA + CJS were markedly older than those without joint symptoms (57.1 years vs 38.7 years) and were more likely to be female (60.5% vs 51.7%), have a high school diploma or less education (59.3% vs 45.4%), and have an annual household income of <$35,000 (66.9% vs 49.0%). Among DDA + CJS respondents, 66.4% were non-Hispanic white, 28.1% were non-Hispanic black, 1.8% were Hispanic, and 5.5% were of other racial/ethnic groups. Because of Medicare's broad coverage, most people in all subgroups were insured.

Respondents with DDA + CJS reported worse health status than did those with NJS (Table 3). Specifically, those in this subgroup reported poorer general health (49.4% vs 8.8%), physical health (53.2% vs 21.3%), and mental health (35.9% vs 29.5%). Furthermore, they tended to have lower physical activity levels (30.9% vs 44.5%), to be more sedentary (29.9% vs 16.0%), and to be overweight or obese (76.3% vs 56.4%). Respondents with DDA and respondents with CJS were more likely to report poor health status than those who reported NJS and IJS.

To illustrate the differences between health status, physical activity, obesity, and sociodemographic factors among subgroup members, we performed multiple logistic regression analyses (Table 4). The adjusted ORs for 4 subgroups (IJS, CJS, DDA − CJS, and DDA + CJS) reflect comparisons with the reference subgroup (NJS). These comparisons revealed significant differences between the subgroups.

- Respondents with DDA + CJS were more likely to have fair to poor health (OR, 10.0), to be physically inactive (OR, 2.7), and to be overweight or obese (OR, 2.5) than were respondents in the NJS group.
- Male respondents were more likely to be overweight or obese (OR, 1.9) than were female respondents.
- Those with less education were more likely to have fair to poor health (OR, 2.7), to be physically inactive (OR, 2.8), and to be overweight or obese (OR, 1.2) than those with some college or more education.

However, the rate of overweight/obesity was high in each of the 5 subgroups: NJS (56.4%), IJS (65.8%), CJS (68.9%), DDA − CJS (73.9%), and DDA + CJS (76.3%) (Table 3). Results from the above regression analyses were controlled for age, educational attainment, race/ethnicity, and household income. Post hoc analyses indicated these same variables did not significantly contribute to the amount of variance explained in the plenary regression model.

Limitations of usual activities as a result of chronic joint symptoms, arthritis, or both (Table 5) were most pronounced among respondents in 3 subgroups: DDA + CJS (54.2%), DDA − CJS (36.3%), and CJS (21.1%). Similar percentages — 51.4% for those with DDA + CJS, 28.3% for those with DDA − CJS, and 24.0% for those with CJS — were observed among those whose ability to work, the type of work performed, or the amount of work performed was adversely affected by arthritis or joint symptoms. Smaller percentages — 24.1% for those with DDA + CJS, 15.6% for those with DDA − CJS,  and 5.9% for those with CJS — indicated having a health problem that "requires use of special equipment such as a cane, wheelchair, a special bed, or a special telephone."

## Discussion

Our study resulted in 2 important observations. First, obesity levels were unacceptably high in all of the study's subgroups: 35% of respondents were overweight, and 27% were obese. The results of a recent study suggest that Mississippians lead the nation in the number of residents who are either overweight or obese and in the number who face obesity-related health risks such as diabetes, hypertension, and rheumatic disorders (12). Mississippi's unacceptably high percentages are consistent with a national trend toward higher obesity rates across several age groups with no evidence of a leveling off, even among older adults (13). Concomitantly, medical costs related to obesity continue to rise; annual expenditures averaged $263 (in 2003 dollars) per Mississippian (12).

The problem of obesity is no less severe among Mississippi's youth. The prevalence of overweight students is higher than the national average: 16% of Mississippi's students are overweight compared to the national average of 12%. An additional 16% are at risk of becoming overweight, compared to the national average of 15%. Mississippi ranks second in the nation for the number of high school students who are overweight (14). Being overweight is a risk factor for osteoarthritis because of the increased load on the joints (15). Obesity (and the comorbid disease of diabetes) has been elevated to the top of the list of the state's most pressing public health concerns (4).

The second important finding is related to noticeable differences between older and younger respondents. In general, older adult Mississippians reported poorer health, less physical activity, and a greater need for medical treatment. Although we anticipated, based on results from external studies (15-17), that the prevalence of arthritis and chronic joint symptoms would be significantly related to sex, race/ethnicity, age, educational attainment, household income, functional disability, and obesity, the magnitude of the differences in these factors related to age was unexpected.

One of the most interesting differences was found between the DDA + CJS and DDA − CJS respondents. Although the mean ages and the number of seniors aged 65 or older in each subgroup were similar, DDA − CJS respondents reported substantially fewer limitations in their ability to conduct their daily activities, including those that were work-related. The difference in disease burden is unlikely to be the consequence of aggressive medical interventions; more likely, it is the result of differing types of rheumatic disease, duration of disease onset, the area of the body affected, or personal lifestyles.

In contrast to the DDA + CJS and DDA − CJS respondents, those in the CJS subgroup were younger (88.9% were aged 18–64 years) and were less likely to be functionally impaired or to have seen a doctor or other health professional for their joint symptoms. Additionally, compared to those with NJS, members of the CJS subgroup were almost 3 times as likely to report fair to poor general health and 2.7 times as likely to be overweight or obese. These findings are consistent with those from an earlier BRFSS study, during which researchers noted that members of the DDA + CJS, DDA − CJS, and CJS subgroups had significantly worse health-related quality of life than those without arthritis and that those with DDA have consistently worse general health than those with only CJS (3). These persistent, unaddressed medical issues indicate that members of these subgroups should be the focus of both prescriptive and preventive medical intervention.

One proven way to minimize the progression of debilitating rheumatic disease is through compliance with an exercise regimen (16-18). Regular exercise can improve the health status and thereby the quality of life for approximately 70 million Americans, including approximately 1 million Mississippians with arthritis or chronic joint symptoms (19). Objective measures of the effectiveness of exercise as a nonmedical treatment among this population have been variously demonstrated (20-24). Exercise (with or without weight loss) is effective in reducing the damaging effects of disease, in certain instances, by as much as 50% (25,26).

Of particular interest to adults with arthritis is the potential for marked improvement in overall functionality through exercise (27). Given the mounting evidence that deconditioned muscles, inadequate motion, and joint stiffness contribute to disease signs and symptoms (28), well-conditioned muscles and muscular balance are, therefore, needed to attenuate impact loads, provide joint stability, and support function and independence. Both muscular conditioning and balance can be achieved through well-designed exercise programs that incorporate training for strength and endurance at functional speeds and in functional patterns (29).

## Conclusion

Health status, physical fitness, and weight control may be substantially improved through increased exercise. However, Mississippians, especially older patients with arthritis or chronic joint symptoms, have historically been reluctant to commit to exercise regimens (30-34). Findings from this study suggest a need to encourage Mississippians with arthritis and chronic joint symptoms to engage in some regular physical activity, which could reduce the damaging effects of disease and improve their health. Increasing the health care resources earmarked for arthritis self-help and physical activity programs is one potential avenue to address the problem.

## Figures and Tables

**Table 1:** Survey Item Responses, by Arthritis-Related Subgroup — Mississippi Behavioral Risk Factor Surveillance System, 2003

**Arthritis-Related Subgroups**	**During the past 30 days, have you had any symptoms of pain, aching, or stiffness in or around a joint?**	**Did your joint symptoms first begin more than 3 months ago?**	**Have you ever seen a doctor or other health professional for these joint symptoms?**	**Have you been told by a doctor you have some form of arthritis, rheumatoid arthritis, gout, lupus, or fibromyalgia?**
IJS	Yes	No	No	No
CJS	Yes	Yes	Yes or No	No
DDA - CJS	Yes or No	No	Yes or No	Yes
DDA + CJS	Yes	Yes	Yes or No	Yes
NJS	No	No	No	No

IJS indicates intermittent joint symptoms; CJS, chronic joint symptoms; DDA - CJS, doctor-diagnosed arthritis without chronic joint symptoms; DDA + CJS, doctor-diagnosed arthritis with chronic joint symptoms; NJS, no joint symptoms.

**Table 2:** Sociodemographic Differences, by Arthritis-Related Subgroup — Mississippi Behavioral Risk Factor Surveillance System, 2003

**Sociodemographic Characteristics**	**IJS**	**CJS**	**DDA-CJS**	**DDA + CJS**	**NJS**
Mean age, y	38.0	42.2	54.7	57.1	38.7
Female, %	46.8	46.3	58.8	60.5	51.7
High school graduate or less, %	44.8	50.2	53.8	59.3	45.4
Annual household income <$35,000 per year, %	52.9	51.2	65.5	66.9	49.0
Health care coverage, %	76.5	76.7	90.5	84.3	80.4
**Race/Ethnicity**					
White (non-Hispanic), %	60.0	66.5	56.2	66.4	59.8
Black (non-Hispanic), %	37.5	28.3	42.1	28.1	34.9
Hispanic, %	1.6	1.5	0.8	1.8	2.2
Other, %	0.9	3.7	0.9	5.5	3.1

IJS indicates intermittent joint symptoms; CJS, chronic joint symptoms; DDA - CJS, doctor-diagnosed arthritis without chronic joint symptoms; DDA + CJS, doctor-diagnosed arthritis with chronic joint symptoms; NJS, no joint symptoms.

**Table 3:** General Health Status, Physical Activity, and Obesity of Respondents, by Arthritis-Related Subgroup — Mississippi Behavioral Risk Factor Surveillance System, 2003

**Health Characteristic **	**IJS (%)**	**CJS (%)**	**DDA - CJS (%)**	**DDA + CJS (%)**	**NJS (%)**
Fair or poor general health	13.6	22.0	36.7	49.4	8.8
Poor physical health prior 30 days	28.4	35.0	41.1	53.2	21.3
Poor mental health prior 30 days	39.2	43.8	36.3	35.9	29.5
Meets recommended physical activity level^a^	44.9	42.8	28.9	30.9	44.5
Insufficient physical activity level^b^	39.8	39.5	36.6	39.3	39.5
Inactive^c^	15.3	17.7	34.5	29.9	16.0
Overweight or obese^d^	65.8	68.9	73.9	76.3	56.4

IJS indicates intermittent joint symptoms; CJS, chronic joint symptoms; DDA - CJS, doctor-diagnosed arthritis without chronic joint symptoms; DDA + CJS, doctor-diagnosed arthritis with chronic joint symptoms; NJS, no joint symptoms.

a Reported at least 30 minutes of moderate activity 5 or more days a week or at least 20 minutes of vigorous activity 3 or more days per week.

b Physical activity reported, but less than the recommended physical activity level.

c No reported physical activity in the prior 30 days.

d Body mass index ≥25 kg/m^2^.

**Table 4:** Demographic Characteristics, General Health Status, Physical Activity, and Overweight or Obesity — Mississippi Behavioral Risk Factor Surveillance System, 2003

**Respondent Characteristics**	Fair to Poor General Health Status OR (95% CI)	Physically Inactive^a^ OR (95% CI)	Overweight or Obese^b^ OR (95% CI)
**Demographics**			
Age ≥65 y	3.5 (2.9-4.1)	3.4 (2.4-3.6)	0.8 (0.7-1.0)
Sex: male	0.7 (0.6-0.8)	0.6 (0.5-0.8)	1.9 (1.6-2.2)
High school diploma or less	2.7 (2.3-3.2)	2.8 (2.3-3.3)	1.2 (1.1-1.4)
Race/ethnicity: black (non-Hispanic)^c^	1.1 (0.8-1.4)	1.9 (1.6-2.3)	1.5 (1.2-1.8)
**Symptoms^d^**			
Intermittent joint symptoms	1.6 (1.0-2.5)	1.0 (0.6-1.5)	1.5 (1.1-2.1)
Chronic joint symptoms	2.9 (2.2-3.8)	1.2 (0.9-1.6)	1.7 (1.4-2.2)
Doctor-diagnosed arthritis without chronic joint symptoms	6.0 (3.7-9.8)	3.3 (1.9-5.9)	2.2 (1.3-3.6)
Doctor-diagnosed arthritis with chronic joint symptoms	10.0 (8.1-12.6)	2.7 (1.1-3.5)	2.5 (2.1-3.0)

OR indicates odds ratio; CI, confidence interval.

a No reported physical activity in the prior 30 days.

b Body mass index ≥25 kg/m^2^.

c Race/ethnicity comparisons were to non-Hispanic whites.

d Reference group was those with no reported joint symptoms.

**Table 5:** Percentage of Respondents With Activity Limitation, by Arthritis-Related Subgroup — Mississippi Behavioral Risk Factor Surveillance System, 2003

**Activity Limitation and Health Care Access Factor**	**CJS (%)**	**DDA - CJS (%)**	**DDA + CJS (%)**
Limited in usual activities because of arthritis or joint symptoms	21.1	36.3	54.2
Arthritis or joint symptoms affect the ability to work, the type of work performed, or the amount of work performed	24.0	28.3	51.4
Requires the use of special equipment	5.9	15.6	24.1
Seen a doctor or other health professional for joint symptoms	47.1	NA^a^	90.8

CJS indicates chronic joint symptoms; DDA - CJS, doctor-diagnosed arthritis without chronic joint symptoms; DDA + CJS, doctor-diagnosed arthritis with chronic joint symptoms.

a Not asked because of the sequencing of the interview questions within the core interview module.
